# Strategic Analysis of the Robotic Surgery Market

**DOI:** 10.7759/cureus.111333

**Published:** 2026-06-22

**Authors:** Matthew Mavandi, Elif B Dilden

**Affiliations:** 1 Department of Medical Education, College of Osteopathic Medicine, Kansas City University, Kansas City, USA; 2 Department of Economics, College of Business and Technology, Helzberg School of Management, Rockhurst University, Kansas City, USA

**Keywords:** healthcare innovation, healthcare technology, medical device market, porter’s five forces analysis, robotic surgery, surgical robotics

## Abstract

Robotic-assisted surgery represents a rapidly growing segment of the US medical device market. This editorial applies Porter’s Five Forces, political, economic, social, and technological (PEST) analysis, and value, rarity, inimitability, and organization (VRIO) analysis, along with qualitative interpretation, to examine the competitive structure of the robotic surgery market. High development costs, extensive healthcare regulation, and limited technological supply have slowed competitive entry, but growing demand has intensified competitive pressure. The market appears to favor established firms because of high switching costs, existing long-term hospital relationships, and surgical familiarity with established robotic platforms. Existing firms may be positioned for continued growth given these structural advantages; however, long-term success depends on their ability to scale efficiently, strengthen hospital relationships, and deliver value in an increasingly competitive, capital-intensive, and innovation-driven environment. Building on these findings, this analysis offers differentiated strategic implications for incumbents seeking to defend market position, established rivals pursuing growth, and well-capitalized entrants identifying viable points of entry into this complex market.

## Editorial

Introduction

Robotic-assisted surgery has become an increasingly important component of modern surgical practice. This editorial examines the evolution, market structure, and competitive dynamics of robotic surgical systems. Understanding these factors is essential for evaluating how these technologies influence surgical adoption and healthcare delivery. Therefore, this editorial evaluates the robotic-assisted surgery market using Porter’s Five Forces; political, economic, social, and technological (PEST) analysis; and value, rarity, inimitability, and organization (VRIO) analysis, with emphasis on competitive dynamics, hospital adoption, and strategic implications for incumbent firms, emerging competitors, and new entrants.

Evolution of Robotic Surgery

Historically, surgeons performed procedures using the open approach, which involves making a wide incision with a scalpel and completing the operation within the body cavity. As technology advanced, laparoscopic cameras were developed to allow surgeons to operate through small incision sites. Demand for minimally invasive laparoscopic surgery grew as patients experienced less pain, shorter hospital stays, and improved outcomes. Naturally, the use of surgical robots evolved from the laparoscopic model. Robotic-assisted surgery has gained substantial adoption in selected surgical fields because of its enhanced visualization, instrument articulation, and ergonomic advantages compared with conventional laparoscopic surgery.

One of the earliest uses of an industrial robot in surgery involved using the PUMA 200 robotic arm (Westinghouse Electric; Pittsburgh, Pennsylvania, USA) in 1985, for needle placement in a CT-guided brain biopsy [1]. Later, in 1992, a surgical team used a ROBODOC surgical system (Integrated Surgical Systems; Sacramento, California, USA) to perform the first robot-assisted human hip replacement [2]. In 1995, Intuitive Surgical created its first robotic surgical prototype, “Lenny,” named after Leonardo da Vinci. The initial Intuitive Surgical two-arm robotic system and one camera holder of the da Vinci operating system (Intuitive Surgical; Sunnyvale, California, USA) began to be used in Europe in 1999 [1]. Intuitive Surgical’s fourth-generation device, launched in 2014, called da Vinci Xi, expanded its clinical use due to improved surgical versatility [3].

Intuitive Surgical’s first-mover advantage enabled the firm to secure substantial early capital investments, which accelerated product development, infrastructure build-out, and market penetration. As demand for robotic-assisted surgery increased, a growing list of competitors entered the market. Medtronic’s Hugo Robotic-Assisted Surgery (RAS) System (Medtronic; Minneapolis, Minnesota, USA) received FDA clearance for minimally invasive urologic surgical procedures in the United States in December 2025 [4]. Similarly, CMR Surgical’s Versius Surgical System (CMR Surgical; Cambridge, United Kingdom) gained FDA marketing authorization through the De Novo pathway in October 2024 [5]. Yet, industry competition has been limited by high manufacturing costs, complex regulatory requirements, and extensive infrastructure demands. As a result, smaller companies are often unable to sustain the long-term capital demands required to successfully compete in this market.

Surgical Robotics Market Structure

This editorial focuses on the US robotic-assisted surgery market. A market definition of a robotic surgery system is a computer-controlled platform designed to enhance precision, control, and visualization [6]. The surgical robotics market remains relatively new, with few major competitors due to its high cost of entry. Intuitive Surgical leads the robotic surgery technology industry through its da Vinci Surgical System. However, the market continues to evolve as diverse robotic systems are adopted clinically. 

Robotic surgery systems can also be highly specialized products offering unique technological features and clinical applications. For example, Stryker’s Mako SmartRobotics (Stryker; Kalamazoo/Portage, Michigan, USA) is used in orthopedic surgery for increased precision [7]. Johnson & Johnson’s Monarch Platform (Johnson & Johnson MedTech/Auris Health; Redwood City, California, USA) and Intuitive Surgical’s Ion endoluminal system (Intuitive Surgical; Sunnyvale, California, USA) are both robotic-assisted bronchoscopy systems used to visualize the airways [8-9]. Globus Medical’s ExcelsiusGPS robotic navigation platform (Globus Medical; Audubon, Pennsylvania, USA) is used in spine and cranial surgery [10].

Strategic framework

This editorial synthesizes publicly available literature, industry reports, regulatory announcements, and peer-reviewed studies related to robotic-assisted surgery. Sources were selected to provide historical, clinical, economic, and strategic context for evaluating the robotic surgery market. Given the editorial and qualitative nature of this analysis, sources were selected narratively based on relevance to the strategic analysis rather than through a systematic review protocol. Conclusions were therefore interpreted cautiously and framed as qualitative assessments rather than empirically derived market measurements.

Porter’s Five Forces

Michael Porter’s Five Forces Framework identifies the forces that threaten the profitability of firms within an industry [11]. These five forces converge to shape how fiercely companies compete [12]. Analyzing the five forces can help companies anticipate shifts in competition, shape how industry structure evolves, and find better strategic positions within the industry [11].

Internal Rivalry

Internal rivalry, also called competitive rivalry, is the most direct and immediate force affecting a company's ability to succeed within an industry. Although Intuitive Surgical is the industry leader, its rivals' strategic choices influence competitive dynamics and firm behavior across the market. For example, one secondary source estimates the da Vinci 5 robot may be priced between $1.8 and 2.5 million; meanwhile, Medtronic’s Hugo may be priced between $0.9 and 1.2 million [13]. Although exact pricing varies by contract, leasing model, service agreement, region, or hospital volume, these estimates suggest that lower-priced platforms may reduce purchasing barriers for some hospitals.

Entry

Hospitals have scrutinized budgets and fixed reimbursement rates for each type of surgery. They must justify spending millions of dollars on technologically advanced equipment. Long-term contracts may discourage or constrain hospitals with strict budgets from purchasing, leasing, or trialing competing robotic surgery systems. Steep experience curves also put entrants at a cost disadvantage. Learning curves include building sustained partnerships with healthcare providers, crafting robots to the surgeon's preference, and gaining access to hospitals. Firms must also invest heavily in research and development and specialized manufacturing infrastructure, which increases capital intensity and reinforces barriers to entry. Moreover, incumbent firms have invested years in meeting stringent regulatory requirements, further elevating entry barriers. These significant barriers to entry deter new entrants.

Substitutes

Substitutes are alternative approaches that meet the same clinical need. For robotic-assisted surgery, the major substitutes are conventional laparoscopic surgery and open surgery. Laparoscopic surgery remains the strongest substitute because it is widely available, familiar, and often less costly than robotic-assisted surgery. For example, one national analysis found that average hospitalization costs for robotic-assisted abdominal operations were $18,300 compared to $16,000 for laparoscopic cases, with robotic procedures yielding only a 2.2% reduction in complications and a 0.7-day shorter length of stay [14]. Therefore, substitutes remain an important competitive force, especially for lower-volume institutions and procedures where robotic surgery does not clearly improve clinical or economic value.

Supplier Power

In the surgical equipment market, accuracy and precision are essential because of the high stakes of patient care. Robotic surgical systems require specialized components, which may include robotic arms, computer chips, imaging systems, endoscopic cameras, software, and surgical instruments. These components require rigorous quality control and regulatory compliance, which can make supplier changes difficult. However, supplier power may vary depending on the degree of component customization and whether the robotic system manufacturer controls parts of its own supply chain. Therefore, supplier power remains a relevant but variable force in the robotic surgery market.

Buyer Power

Robotic systems may also be acquired or operated by ambulatory surgery centers, outpatient surgical facilities, integrated healthcare systems, and other appropriately licensed institutions. These purchasing organizations may perceive advanced robotic systems as high-quality, state-of-the-art technologies that signal surgical capability, support recruitment, and may improve outcomes in selected procedures. As more robotic platforms enter the market, buyers may gain additional leverage when negotiating pricing, financing, service agreements, and platform support. Buyer power may also increase through leasing and flexible acquisition models that reduce upfront capital barriers.

Implications of the Five Forces

The US robotic surgery market is shaped by increasing competitive rivalry, rising buyer power, substantial entry barriers, procedure-dependent substitutes, and variable supplier power. Competitive rivalry and buyer power are central forces as robotic platforms compete on pricing and hospital relationships, while buyers gain leverage through flexible purchasing models and increased robotic surgery platform options. Entry has been consistently difficult because firms must overcome high development costs and regulatory requirements. Laparoscopic or open surgery remains a clinically viable substitute, often at lower cost. Supplier power may vary depending on component specialization and supply-chain control.

**Figure 1:**
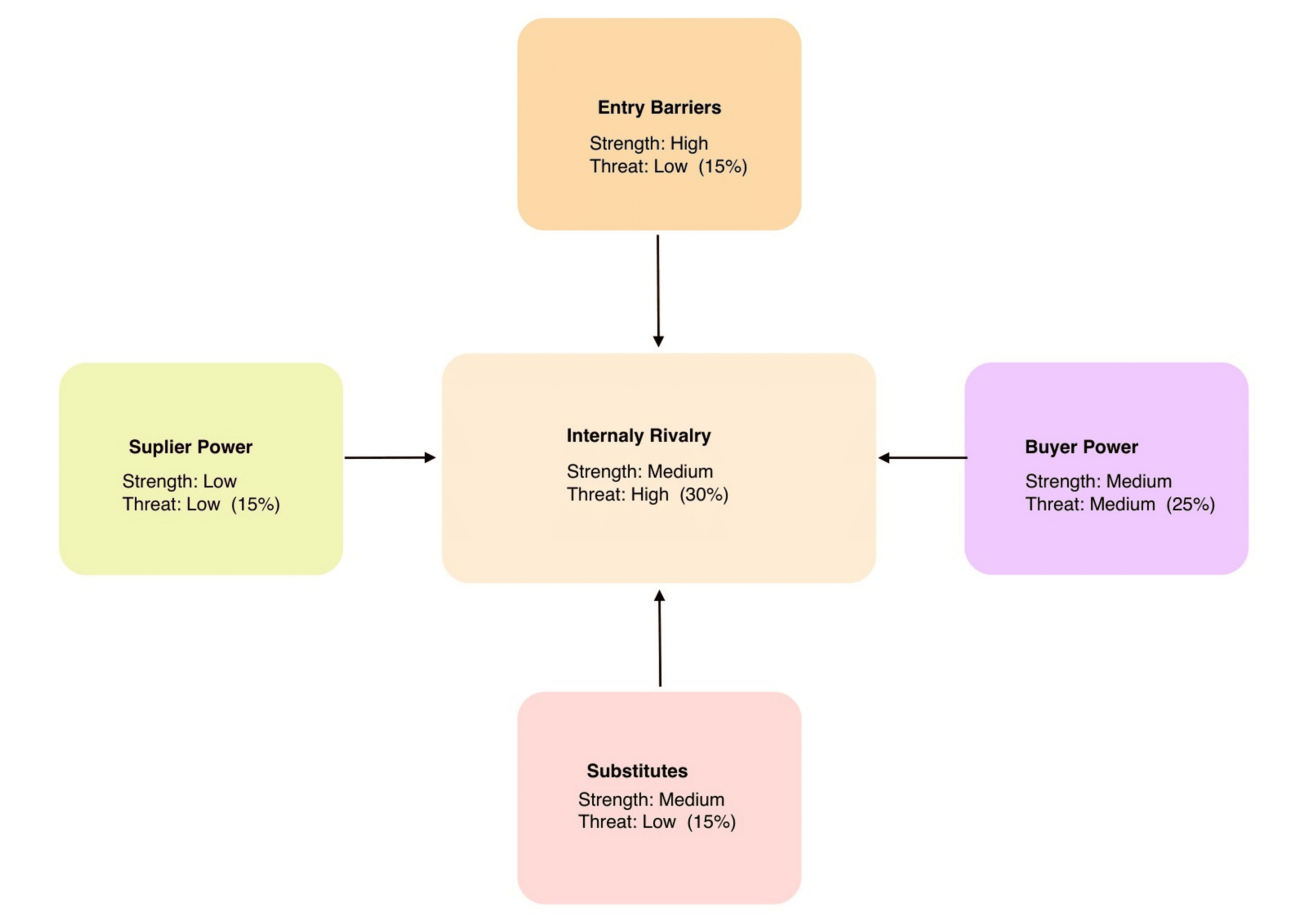
Porter’s Five Forces analysis of the robotic surgery industry. The strength, threat, and weight values were assigned by the authors based on qualitative assessment of the robotic surgery market and are intended as illustrative estimates rather than empirically derived measurements. Created with Pages by the authors. No artificial intelligence tools were used to create this image.

Figure 1 provides a visual representation of Porter’s Five Forces framework as applied to the robotic surgery industry. To reflect the relative strategic significance of each force in this context, the authors assigned qualitative strength, threat, and percentage weight values based on their assessment of each force’s likely impact on long-term industry profitability. “Strength” refers to the relative intensity of each competitive force in the robotic surgery industry. “Threat” refers to the threat to profitability. “Weight” refers to the relative importance assigned to each force in estimating its overall effect on industry profitability. Internal rivalry received the highest weight at 30% because of accelerating competitive entry and its direct implications for pricing power and market share. Buyer power received the second-highest weight at 25%, reflecting the growing negotiating leverage of hospital systems as credible alternatives proliferate. Entry barriers, substitutes, and supplier power were each weighted at 15% because of their comparatively stable influence on industry dynamics.

Political, economic, social, and technological (PEST) analysis

Political factors influence the market primarily through regulation, reimbursement, and healthcare spending priorities. Changes in healthcare spending and variable reimbursement rates may constrain hospital budgets and slow the adoption of high-cost surgical technologies. Publicly traded robotic surgery companies must also respond to shareholder expectations. Robotic surgery companies may also engage with policymakers or advocacy groups on reimbursement and regulatory requirements. The robotic surgery industry is also heavily influenced by public regulatory agencies such as the FDA.

Economic factors also shape adoption. Robotic surgical systems are high-capital-cost technologies and may be vulnerable to budgetary restrictions when hospital administrators perceive limited incremental clinical or financial value. Hospitals may delay or avoid purchasing high-cost robotic surgical systems when budgets are limited or reimbursement is uncertain. High platform cost may have slowed adoption in resource-constrained settings such as rural hospitals and safety-net institutions. Policymakers and hospital administrators should consider whether expanded access to robotic surgery translates into equitable patient outcomes across incomes and geographic lines. Additionally, large-scale economic and public health disruptions, such as financial recessions or global pandemics, could put robotic surgery companies in financial distress.

Interest in robotic surgery has increased because robotic surgery is often associated with perceived technological advancement and faster recovery times in selected procedures. Robotic surgery may be more readily adopted by trainees and surgeons who gain exposure to these platforms during residency or fellowship. Meanwhile, adoption by established surgeons may vary depending on individual preference, institutional access, credentialing requirements, and perceived clinical value. These adoption factors should be considered when evaluating entry into this competitive market.

To succeed in this technologically advanced industry, firms must continually push toward innovation. These innovations should meet the evolving demands of hospital systems and surgeons. However, innovative technologies need to be accepted by governments, patients, and communities before widespread use. Adoption of newer robotic surgical innovations can encounter resistance, which may include cybersecurity, artificial intelligence (AI) oversight, and data governance concerns. Effectively addressing this resistance through targeted training, support, and engagement is critical to the successful implementation.

Competitive Landscape

The PEST analysis and five forces analysis illustrate the rising competition within the robotic surgery industry. Regulatory requirements, reimbursement uncertainty, hospital capital budgets, and surgeon training patterns affect the ability of firms to enter and compete in this market. External economic pressures can increase buyer power and intensify competitive rivalry. Technological advances may also increase supplier power and hospital demand. Together, these frameworks highlight the increasing competitive pressure within the market.

Industry strategy

Diversification can create value, reduce risk, and generate revenue growth. However, companies must be cautious as diversification has also caused more value destruction than almost any other type of strategic initiative [15]. Vertical integration describes a company’s ability to control its manufacturing, supply chain, and distribution. Vertical integration helps firms reduce their dependence on other companies and improve profit margins. Robotic surgery firms can utilize economies of scope by bundling services, including maintenance and surgical supplies.

Leading firms should invest in competency-based training programs that help surgeons and trainees use robotic platforms safely, effectively, and appropriately. Exposure during residency and fellowship can shape surgeon familiarity, comfort, and workflow habits. If manufacturers choose to support residency, fellowship, or simulation events, these relationships should focus on patient safety and technical training, while maintaining transparency, independent educational oversight, and clear conflict-of-interest safeguards [16].

Further, AI technologies are rapidly permeating every sector of the economy, transforming how businesses operate, analyze data, and deliver value. Integrating AI into robotic surgical systems could provide strategic advantages, potentially strengthening their competitive position, differentiating their products, and accelerating the adoption of robotic surgery in hospitals worldwide.

There is a strong demand to globalize surgical robotic products as medical professionals and healthcare systems seek to advance their surgical capabilities. However, navigating country-specific regulations and staffing hospitals with qualified medical representatives presents significant logistical challenges. Overcoming these formidable regulatory and operational barriers requires substantial capital investment, but successful market entry can yield considerable returns.

VRIO analysis of Intuitive Surgical

VRIO stands for valuable, rare, costly or difficult to imitate, and whether the firm is organized to capture or exploit the value generated by that resource. The VRIO framework is used to identify and evaluate firm resources that lead to sustained competitive advantage. Competitive advantage refers to a firm's ability to outperform its rivals [15]. Once firm resources have been identified, firms can begin to devise an effective company strategy. The VRIO framework may help explain why Intuitive Surgical is currently performing above industry average.

First, Intuitive Surgical’s nationwide implementation of da Vinci robotic surgery systems is one of its most valuable resources. Its installed base of thousands of da Vinci systems is valuable because it generates recurring revenue through service agreements, disposable instrument utilization, and regular maintenance. This embedded network of multiple generations of da Vinci platforms is difficult to imitate because hospitals may face high switching costs, workflow disruptions, and additional surgeon training requirements when adopting a competing platform. Overall, this existing installed base is likely one of Intuitive Surgical’s greatest resources and may help explain its sustained competitive advantage.

Next, surgeons and staff have already become familiar with da Vinci systems and may influence training pathways and institutional purchasing preferences. When authorized by institutional policy, Intuitive Surgical representatives may be present in the operating room to provide product-related support. This setting may allow company representatives to observe product use and receive real-time feedback from surgeons.

Finally, through first-mover advantage, Intuitive Surgical has developed and improved its design that may be difficult for competitors to imitate. Although da Vinci systems are complex engineering technologies, their customer interface is intended to be user-friendly. However, usability alone does not determine surgical outcomes. Effective use of robotic systems depends on surgeon training, procedural complexity, patient characteristics, operating-room workflow, staff experience, and institutional support.

Despite these advantages, Intuitive Surgical also faces important vulnerabilities. High platform costs may limit adoption. The da Vinci platforms are limited to select surgical procedures, whereas other robotic systems or newer robotic designs may offer alternative applications in surgery. Equipment setup, turnover, cleaning, disinfection, sterilization, and storage requirements may also create workflow challenges and require specialized training.

Overall, these resources and capabilities illustrate why Intuitive Surgical has been able to perform above industry average and maintain a leading market position. Intuitive Surgical’s existing network, technical customer support systems, and surgeon engagement have led to its successful implementation of this new technology. However, its long-term position will depend on continued product development, demonstrated clinical value, cost-effectiveness, hospital affordability, surgeon adoption, and its ability to respond to emerging competitors.

Implications for managers of incumbent firms

The primary strategic objective for incumbent firms should be to defend and deepen existing competitive advantages. These firms already have a loyal customer base, strong hospital relationships, and leverage extensive data to guide product development. An effective strategy is to encourage existing hospital customers to purchase additional robotic surgery systems and collaborate with administrators to improve operating room workflow efficiency. Incumbents should continue embedding their platforms into residency, fellowship, and simulation environments. Additionally, incumbents must continue to deliver innovative and convenient solutions or risk losing customers to rising firms that are eager to capture market share.

Implications for managers of rising competitors

Established competitors may benefit from optimizing operating room efficiency or differentiating their products through specialty-specific applications. These firms must also invest heavily in training and surgeon engagement. Partnerships with teaching hospitals, surgical subspecialty societies, and early-adopting surgeons can help rivals establish credibility and gradually build user familiarity. Utilizing a targeted strategy that aligns product capabilities with specialty-specific surgical domains or geographic markets allows rivals to scale more efficiently and avoid direct confrontation with dominant incumbents.

Implications for managers of well-capitalized new entrants

New entrants must be prepared to invest heavily for an extended period before achieving profitability. Success in surgical robotics requires substantial upfront capital, sustained capacity to overcome complex regulatory barriers, and the development of long-term relationships with hospitals and surgeons. Firms that underestimate these demands risk premature exit or strategic retreat. These well-capitalized new entrants could focus on niche markets, specialized procedures, or complementary technologies to overcome brand loyalty. Acquisition-led entry may provide a faster path to market entry by giving firms access to an already authorized product, an existing installed base, and surgeon trust, rather than launching an entirely new product. However, new devices, major product modifications, or additional surgical indications may still require separate regulatory review.

Conclusion

The surgical robotics industry is at an inflection point, characterized by intensifying competitive entry, rising buyer power, and accelerating technological differentiation. Existing companies within the industry may be well-positioned for sustained long-term growth, but they must continually adapt to these competitive forces. A critical and underappreciated source of durable competitive advantage lies in surgical training ecosystems. Firms that invest in transparent, competency-based training for emerging surgeons and resident trainees may shape long-term platform familiarity and institutional adoption decisions. Firms that proactively strengthen hospital partnerships and expand into international markets may be better positioned to capture long-term value. Ultimately, firms that align strategic decisions with evolving customer expectations and effectively navigate institutional constraints will be more likely to lead the next era of surgical robotics. This editorial is subject to inherent limitations because it relies on publicly available market data and qualitative strategic interpretation; future research employing quantitative market analysis would further substantiate these findings.
